# The Middlesex Hospital's Exhibit

**Published:** 1921-02-19

**Authors:** 


### A MEMORABLE EXHIBITION.

### THE MIDDLESEX HOSPITAL'S EXHIBIT.

At Olympia, by both the written and spoken word, the Middlesex is putting forward a short account of research in medicine, and the main object of its actual exhibit is summed up as follows: Many people are intimate with the work of hospitals, but only a few are aware of the importance and far-reaching effects of the work done at those great institutions which have medical schools and research departments attached to them. The truly scientific education of medical men received but scanty popular support in this country until the war made everyone realise that medical education is a great public and national concern. There is no doubt that, in this respect, the highest university standards must be reached, but exactly what these standards imply is still not fully appreciated by the public. The Middlesex Hospital has chosen to illustrate it chiefly in connection with the methods of a laboratory health service, as devoted either to the investigation of an individual patient or of a disease as a whole. They are to be congratulated upon their achievement.

The whole has been arranged by the professors of experimental pathology, pathology, physics, and physiology, the lecturers on biology and chemistry, and the junior physician. A series of lectures upon fluorescence, the ultra-violet rays, *x*-rays, and radium is being given by Professor Lazarus-Barlow, M.D., F.R.C.P.; a series on chemical research in relation to general medicine, industrial health, and pharmacology, by W. B. Tuck, D.Sc.; and an excellent printed guide to the whole exhibit is obtainable at the stand.

It is impossible in a note adequately to outline the wealth of material open to inspection, and to the specialist some of it is already well known; but the point is that just those things are shown and explained by the hospital workers that convey the essential public message.

The whole process is shown in detail by which the study of tumours—"growths," so called—is

**A Memorable Exhibition—(*continued*).**

carried out and how sections of them, $\frac{1}{5000}$ in. or less in thickness, are obtained, and then with the microscope examined at a magnification of eight hundred or more diameters. Benches are in full working order, showing the examination of patients' blood for many kinds of disease. All manner of disease-producing parasites and bacteria are actually shown to the visitor, both under the microscope and alive under artificial—but safe—conditions. The complex modern electrical methods of examining the functions of a patient's heart are demonstrated. One sees complete outfits showing how *x*-rays are actually employed in medicine, and a remarkable series of *x*-ray pictures of disease. Microphotographs of objects almost unimaginably small are actually obtained. A host of chemical processes are open to inspection, not the least interesting being those by which such poisons as arsenic, cocaine, and strychnine are detected in the human body. In the physiological section experiments are being performed, so that the onlooker may actually see a drug take its effect on living tissues and record the result instrumentally. Radium is there in process of checking the growth of living cells. And, last but by no means least, the staff in charge of the exhibit are untiring in their efforts to interest and instruct. Their names we must, at request, withhold, but the lay visitor may rest assured that fame in the research world is not unknown among them.

